# An atypical expression of core α-Dystroglycan and Laminin-α2 in skin fibroblasts of patients with congenital muscular dystrophies

**DOI:** 10.1007/s11033-023-08500-7

**Published:** 2023-06-15

**Authors:** Sahar Sabry, Mahmoud Y Issa, Mohamed S Abdel-Hamid, Noura R Eissa, Sherif F Abdel-Ghafar, Mona M Ibrahim, Maha S Zaki

**Affiliations:** 1 Biochemical Genetics Department, Human Genetics and Genome Research Institute, National Research Centre (NRC), Cairo, Egypt; 2 Clinical Genetics Department, Human Genetics and Genome Research Institute, National Research Centre (NRC), Cairo, Egypt; 3 Medical Molecular Genetics Department, Human Genetics and Genome Research Institute, National Research Centre (NRC), Cairo, Egypt

**Keywords:** Congenital muscular dystrophies, Skin fibroblasts, Core α-dystroglycan, laminin-α2, Glycosylation.

## Abstract

**Background:**

Congenital muscular dystrophies (CMDs) result from genetically inherited defects in the biosynthesis and/or the posttranslational modification (glycosylation) of laminin-α2 and α-dystroglycan (α-DG), respectively. The interaction between both proteins is responsible for the stability and integrity of the muscle cell. We aimed to study the expression profiles of both proteins in two classes of CMDs.

**Subjects and methods:**

Whole-exome sequencing (WES) was done for four patients with neuromuscular manifestations. The expression of core α-DG and laminin-α2 subunit in skin fibroblasts and MCF-7 cells was assessed by western blot.

**Results:**

WES revealed two cases with nonsense mutations; c.2938G > T and c.4348 C > T, in *LAMA2* encodes laminin-α2. It revealed also two cases with mutations in *POMGNT1* encode protein O-mannose beta-1,2-N-acetylglucosaminyltransferase mutations. One patient had a missense mutation c.1325G > A, and the other had a synonymous variant c.636 C > T. Immunodetection of core α-DG in skin fibroblasts revealed the expression of truncated forms of core α-DG accompanied by reduced expression of laminin-α2 in POMGNT1-CMD patients and one patient with LAMA2-CMD. One patient with LAMA2-CMD had overexpression of laminin-α2 and expression of a low level of an abnormal form of increased molecular weight core α-DG. MCF-7 cells showed truncated forms of core α-CDG with an absent laminin-α2.

**Conclusion:**

A correlation between the expression pattern/level of core α-DG and laminin-α2 could be found in patients with different types of CMD.

**Supplementary Information:**

The online version contains supplementary material available at 10.1007/s11033-023-08500-7.

## Introduction

**Fig. 1:**
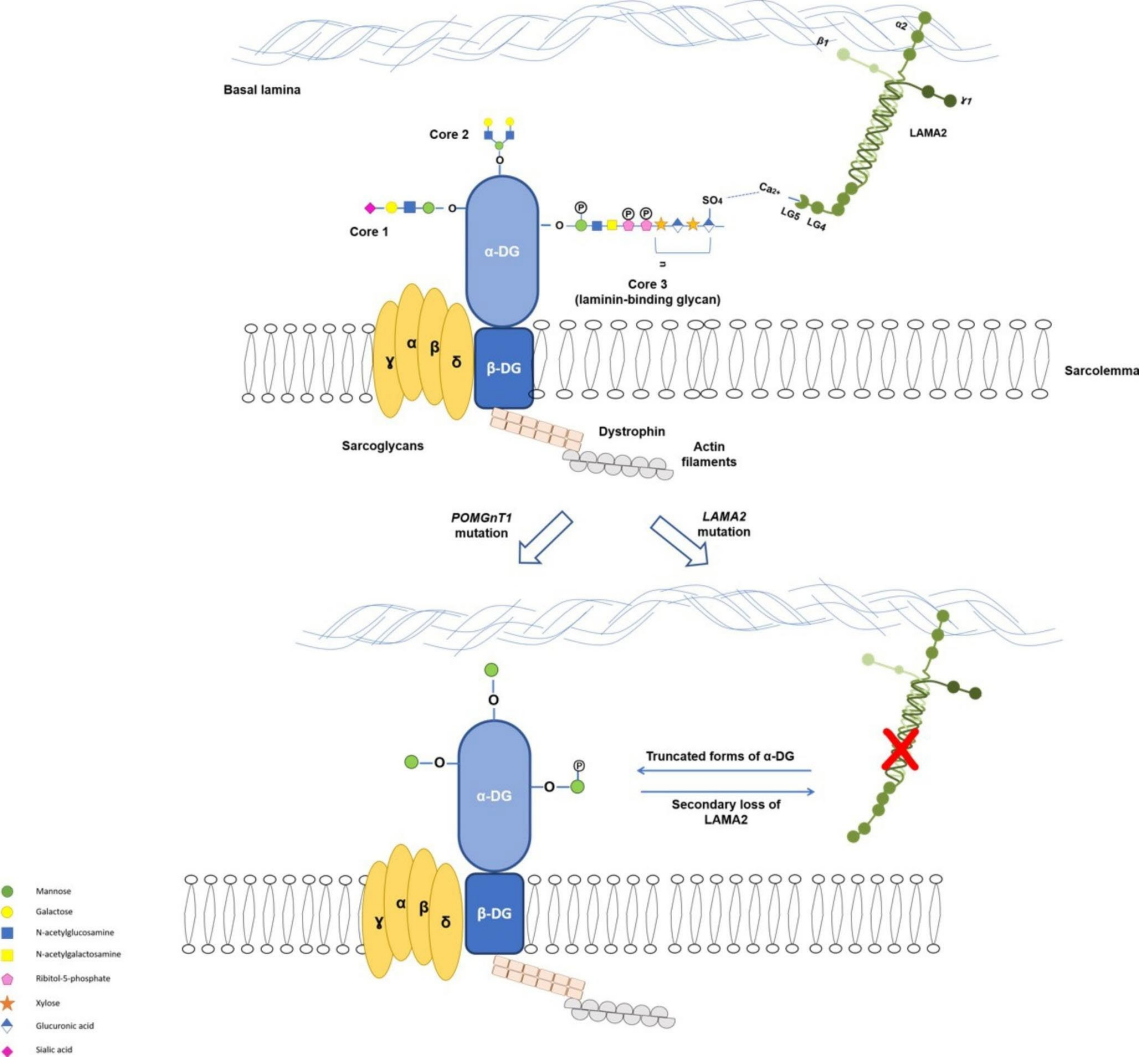
A cartoon representation of the O-mannosyl glycan mediated interaction between α-DG and laminin-α2. The α-DG/laminin-α2 complex forms a physical link between the basal lamina and the cytoskeleton (top). Hypothesized explanation of the observed abnormal expression patterns of α-DG/laminin α2 complex in POMGNT1- and LAMA2-CMD patients (bottom). Incomplete O-mannosyl glycan biosynthesis led to dissociation of the α-DG/laminin-α2 complex and secondary loss of laminin-α2

In mammalian cells, dystroglycan-associated glycoprotein (DAG)/laminin-α2 (merosin) interaction acts as a physical link between the cytoskeleton and the basement membrane (Fig. 1) [1]. Dystroglycan is a glycoprotein that is made up of two subunits (α and β). Alpha-DG carries a unique type of glycans called O-mannosyl glycans (cores 1, 2, and 3) (Fig. 1). Core 3 on α-DG interacts with the laminin-α2 G-like domain 5 (LG5) in the basal lamina [2].

Congenital Muscular Dystrophies (CMDs) have a spectrum of severity that is very broad. It involves mainly the muscles and could extend to include the brain and eyes. Several types of CMDs result from a perturbed interaction between DAG and laminin-α2 interaction [3].

We aimed in this work to study the expression of core α-DG/laminin-α2 in skin fibroblasts derived from patients with CMD. Patients with mutations in *POMGNT1* encodes protein O-mannose beta-1,2-N-acetylglucosaminyltransferase (POMGNT1) enzyme and mutations in *LAMA2* encodes laminin-α2 protein were included in this work.

## Subjects and methods

This work was approved by the Research Ethics Committee of the National Research Centre (registration number 19–254) according to the “World Medical Association Declaration of Helsinki”. Written informed consent was obtained from the patient’s legal guardians.

## Subjects

Four patients with signs and symptoms of CMD were included in our study. All the data of the clinical investigations performed for the included cases are presented in Table 1. The images of MRI of the included cases are shown in supplementary figure (SF 1).

**Table 1:** Data of clinical data of CMD patients

	Patient 1	Patient 2	Patient 3	Patient 4
**Age (years)**	1y	5y	4y	8y
**Sex**	M	M	F	M
**Age of presentation**	9 m	3y	Not reported	5y
**CPK (IU/L)** **N.R.** **39–308 IU/L (males)** **26–192 IU/L (females)**	1250	1225	850	2140
**CNS manifestations**	- Delayed milestones. - History of weak suckling. - Delayed neck support and sitting. - Delayed recognition of parents. - Severe mental retardation	- Delayed motor milestones. - Could sit alone but could not stand or walk. - Hypotonia and hyporeflexia. - History of a similarly affected female sib.	- Developmental delay. - Seizures. - Hypotonia and hyporeflexia.	- Delayed milestones of motor development. - Could sit alone but could not get up from lying position. - Hypotonia and hyporeflexia. - Severe mental retardation
**Cardiac manifestations**	No	No	No	Tachycardia
**Muscle MRI**	Not done	Not done	Not done	Not done
**Eye examination**	Normal	Not done	- Microphthalmia and nystagmus. - Abnormal response of ERG and VEP tests.	Not done
**Brain MRI**	Cobblestone complex, Z shape brain stem.	Deep white matter dysmyelination periventricular.	Cobblestone complex with white matter affection.	Deep white matter dysmyelination periventricular.
**EMG**	Myopathic pattern	Myopathic pattern	Myopathic pattern	Myopathic pattern

## Methods

### Whole exome sequencing (WES)

A solo whole-exome sequencing was performed for both patients using Sure Select Human All Exome 50 Mb Kit (Agilent, Santa Clara, CA, USA) and sequenced using Illumina NovaSeq 6000 (Illumina, San Diego, CA, USA). Identified variants were evaluated concerning their pathogenicity/deleteriousness using PolyPhen_2, SIFT, and Mutation Taster tools.

### Cell culture

Skin fibroblasts derived from a healthy subject and the patients’ skin biopsies and MCF-7 cells were cultured under standard conditions and proteins were extracted then quantified according to the Lowry method.

### Western blot of core α-DG and laminin-α2 proteins

Thirty-five micrograms of protein extracted from skin fibroblasts and MCF-7 cells were loaded on SDS-PAGE gel and then transferred onto nitrocellulose membranes. Protein bands were revealed by a chemiluminescence reaction using ECL (Novex, Invitrogen, Thermo Scientific, US). The membranes were probed with anti-α-DG (Abcam ab 103,905, Cambridge, UK), and anti-laminin-α2 ((B-4): sc-55,605, Santa Cruz, USA), followed by incubation with the secondary antibodies. Beta-actin was used as a housekeeping protein ((C-4): sc-47,778, Santa Cruz, USA).

## Results

### Mutations detected by WES

All the mutations reported in our work had a homozygous pattern.

Patient 1 had a previously reported missense mutation in exon 16, c.1325G > A (p.R442H) of *POMGNT1*. This mutation (rs150877512) resulted in changing the physicochemical properties at the active site of POMGNT1 enzyme (SF 2a). Patient 3 had also a previously reported mutation in exon 6 of *POMGNT1*; c.636 C > T (p.F212=). This mutation was reported under the SNP ID rs190057175 affecting the carbohydrate-binding stem domain of the enzyme (SF 2a). Both mutations were confirmed by Sanger sequencing (SF 3).

The other two patients had nonsense mutations in *LAMA2*. Patient 2 had a novel nonsense mutation in exon 21; c.2938G > T (p.Glu980*), that resulted in a premature stop codon; mapped to the 10^th^ laminin-EGF-like domain (SF 2b). Patient 4 had a previously reported mutation in exon 30; c.4348 C > T (p.R1450*). It was mapped to the 15^th^ laminin-EGF-like domain of the protein, (rs200923373) (SF 2b).

### Core α-DG and laminin-α2 abnormal expression in skin fibroblasts and MCF-7 cells

**Fig. 2:**
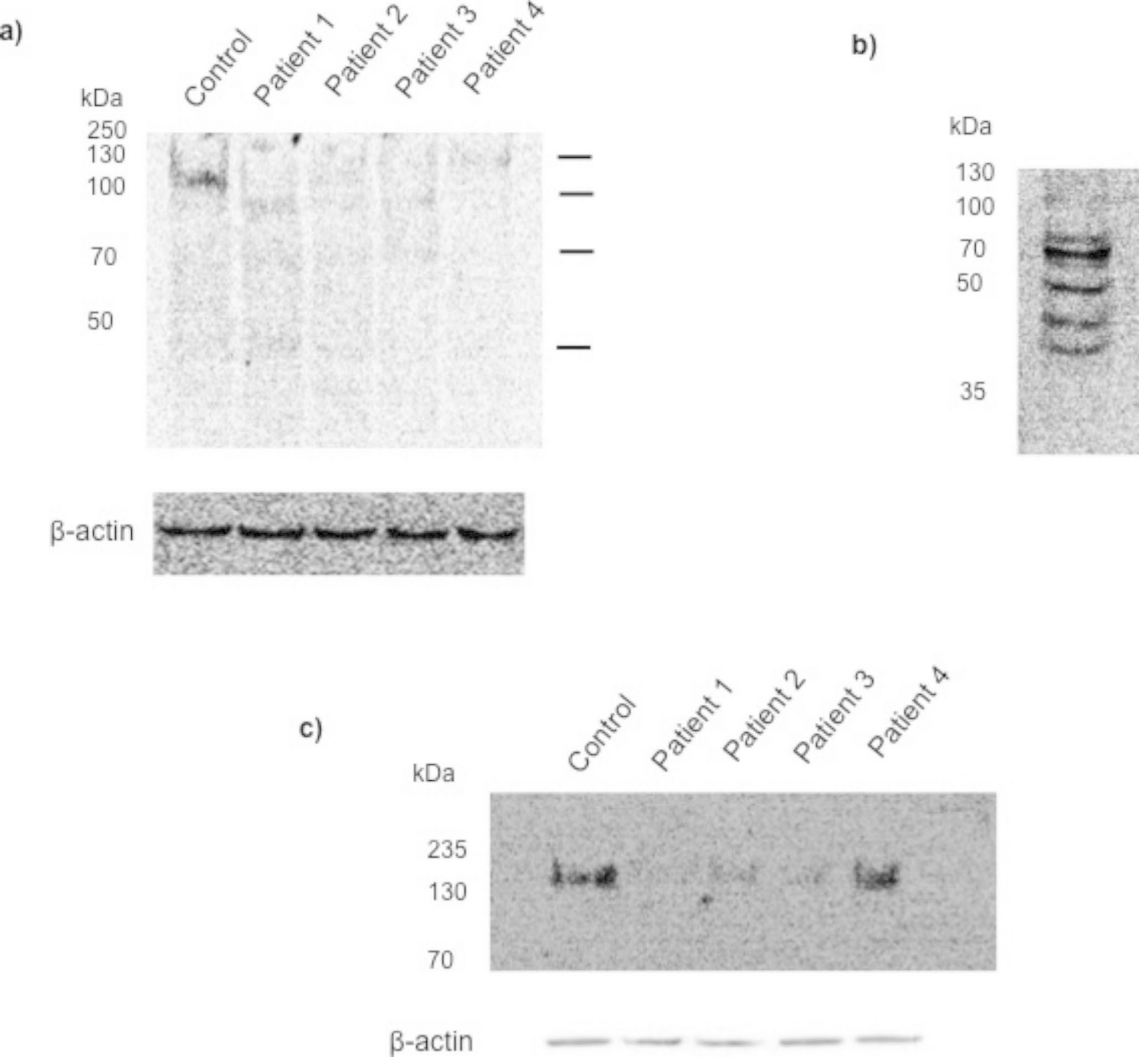
Western blot of core α-DG in (a) skin fibroblasts of patients with POMGNT1-CMD (patients 1 and 3) and LAMA2-CMD (patients 2 and 4) and, (b) MCF-7 cells. C) Western blot of laminin-α2 in skin fibroblasts of patients with POMGNT1-CMD (patients 1 and 3) and LAMA2-CMD (patients 2 and 4). Β-actin was used as a loading control

The immunodetection of core α-DG in the control sample showed a band at a molecular weight (MWt) of 120 kDa. Two bands with lower intensities at MWt 70 and 43 kDa were observed (Fig. 2a). The quantification of the intensities of the bands of core α-DG is shown in SF 4a. The samples from patients 1, 2, and 3 showed bands at an approximate MWt of 100, 70, and 43 kDa. Patient 1’s sample had the highest intensities compared to the other patients’ samples. The sample derived from patient 4 showed one band with a low intensity at MWt > 130 kDa. A multiple bands profile of core α-DG was observed in MCF-7 cells (Fig. 2b). A band at MWt 70 kDa with the highest intensity was observed. Four bands at approximate MWt 80, 55, 45, and 40 kDa were observed. MCF-7 cells showed a reduced intensity of a 120 kDa band was detected. The immunodetection of laminin-α2 revealed a band at MWt > 200 kD in all the samples with different relative intensities (Fig. 2c). The quantification of the intensities of the bands of laminin-α2 subunit is shown SF 4b. The control and patient 4 samples had comparable intensities. Patient 1’s sample showed a higher intensity of laminin-α2 band followed by patient 3 then patient 2. No bands of laminin-α2 protein were detected on separate immunoblotting performed on MCF-7 cell lysate.

## Discussion

Congenital muscular dystrophies (CMDs) are classified into two major classes according to the undelying molecular defects. They either result from the defects in the biosynthesis of a structural protein or the posttranslational glycosylation of α-dystroglycan (also known as dystroglycanopathies) [4]. Our study involved two cases with a dystroglycanopathy (POMGNT1-CMD) (patient 1 and patient 3). They had two previously reported mutations in *POMGNT1* [5]^,^[6]. Two cases with a defective biosynthesis of the structural protein laminin-α2 (LAMA2-CMD) were also included in our study. One case had a reported *LAMA2* mutation (patient 4) [7] and the other case (patient 2) had a novel one. A perturbed interaction between laminin-α2 and α-DG is one of the major underlying causes of neuromuscular signs and symptoms observed in the CMD patients [3]^,^[8]. Unlike most of the studies on CMD that were performed using muscle biopsies as diagnostic samples, we assessed the expression pattern of the physical link-constituting proteins in skin fibroblasts of the CMD cases. Skin fibroblasts have been implemented as an informative and less invasive sample in comparison with muscle biopsy [9]. In our study, the expression levels of the truncated forms of core α-DG were proportional to those of laminin-α2 in POMGNT1-CMD patients and in one case with LAMA2-CMD (patient 2). It has been reported that a partial expression of laminin-α2 was not accompanied by any change in core α-DG expression in a case with LAMA2-CMD [10]. A secondary loss of laminin-α2 has been reported in patients with LARGE- and FRP-CMD with a hypoglycosylation of α-DG [11]^,^[12]. One would expect to find lower levels of the truncated core α-DG in POMGNT1-CMD compared to LAMA2-CMD given that the biosynthesis and glycosylation process of α-DG should be normal in the latter cases. Surprisingly, our POMGNT1-CMD patients had relatively more levels of the truncated core α-DG when compared with LAMA2-CMD (patient 2). They also had relatively more levels of laminin-α2. The second case of LAMA2-CMD (patient 4) showed an overexpressed laminin-α2 protein accompanied with the presence of a form of core α-DG with a molecular weight that was higher than the normal one. It has been reported that missense mutations localized to the N-terminal (5′ region) of *LAMA2* often result in a reduced or a near-normal expression of laminin-α2 protein [13]. This overexpression might be explained by the activation of an alternative promoter [14]. It has been reported that the N-terminus nonsense and structural deletion were associated with a residual protein expression. The alternative promoter seems to produce detectable protein levels [14]. Some compensatory mechanisms for laminin-α2 deficiency were proved to result in an up-regulation of the expression of laminin-α1, -α4, - α5, and mini-agrin (which have the same α-DG-binding ability). The detected α-DG with a molecular weight more than normal in patient 4 might be a truncated form of α-DG bound to a fragment of one of the up-regulated laminin isoforms.

We found a few differences in some clinical signs observed could be correlated with the expression patterns of core α-DG/laminin-α2 complex. For instance, patient 3 had lower expression levels of core α-DG/laminin α2 complex, and her clinical picture involved seizures and eye involvement, which were not observed in the other POMGNT1-CMD patient. Similarly, tachycardia was observed in patient 4 with LAMA2-CMD while patient 2 patient did not show any cardiac manifestations. This might have resulted from the characteristic expression pattern of the observed core α-DG/laminin-α2 complex in patient 4.

Unlike our findings, some reported patients with POMGNT1-CMD who had a reduced expression level of core α-DG; they had a normal expression level of laminin-α2, while other cases of dystroglycanopathies showed a relatively reduced leves of laminin-α2 [15]. Moreover, neural stem cells and animal models with *POMGNT1*^*−/−*^ showed a hypoglycosylated α-DG and a normally expressed laminin-α2 [16]^,^[17].

In order to test the hypothesis that there is a direct correlation between the expression of core α-DG and that of laminin-α2, we used the MCF-7 cell line as a model of defective biosynthesis of the laminin-α2-binding glycans of α-DG. In 2020, Pei J. Lu and colleagues have implemented MCF-7 cells as a system to study the effect of ribitol as a treatment of cancer cells proliferation and metastasis. Ribitol was found to increase the biosynthesis of the laminin-binding matriglycan on the α-dystroglycan [18]. It has been reported that metastatic cancerous cells derived from epithelial tissues have a defective biosynthesis of laminin-binding glycans of α-DG [19]. It was found that the absence of the laminin-binding glycans on α-DG plays a crucial role in the detachment of cancerous cells from the basement membrane and cancer progression [20]. We looked over the expression profile of core α-DG and laminin-α2 expression in MCF-7 cells. The expression of core α-DG/laminin-α2 complex in MCF-7 cells was comparable to our CMD cases. On one hand, and apart from the difference in the relative intensities, we found that the bands of core α-DG found in our CMD patients had almost the same molecular weights as the MCF-7 cells. On the other hand, laminin-α2 was not detected by immunoblotting in MCF-7 cell lysate. The observed absence of laminin-α2 subunit might have resulted from its release to the extracellular space or its degradation as a consequence of the disruption of the physical link with the truncated α-DG.

From the previous findings, we hypothesize that the interaction between core α-DG and laminin-α2 is crucial for stabilizing both proteins. The disruption of this protein-protein interaction might be the underlying molecular mechanism of the similar muscle manifestations observed in most types of CMDs. Future research should be extended to involve different types of CMD to study the expression profiles of both interacting proteins. The limitation of this work involves the small number of the studied cases of CMD. A large-scale study that includes more patients with CMD would be useful to perform the statistical analysis to provide the significance of the observed proteins expression among the study group.

## Electronic supplementary material

Supplementary Material 1

## Data Availability

All data generated or analysed during this study are included in this published article [and its supplementary information files].
