# Supplementary material for: An atypical expression of core α-Dystroglycan and Laminin-α2 in skin fibroblasts of patients with congenital muscular dystrophies
Source: Mol Biol Rep. 2023 Jun 15;50(8):6373–9. doi: 10.1007/s11033-023-08500-7 (PMC10374774; doi:10.1007/s11033-023-08500-7)
Supplement: Supplementary file 1 — Supplementary Material 1 [file 11033_2023_8500_MOESM1_ESM.docx]

**SF1** MRI images of the included patients. A) Patient 1 MRI images showing 1. Midsagittal T2WI MRI showing Abnormally hypoplastic brain stem (Z-Shaped), cerebellar hypoplasia, abnormal cortical gyration, dilated lateral Ventricle, 2. Axial View T2WI showing flat pons with abnormal gyration of the occipital cortex, 3. Axial View T2WI shows abnormal cortical gyration, white matter abnormal myelination, and dilated lateral ventricles. B) Patient 2 MRI images showing 1. Axial T2WI showing extensive white matter affection in inpatient with LAMA2 mutation, 2. Coronal T2WI shows abnormal white matter with abnormal cortical gyration in the form of polymicrogyria. C) Patient 3 showing 1. Axial T2WI MRI showing white matter changes with dilated lateral ventricles and abnormal cortical gyration, 2. Axial T2WI MRI showing hypoplastic pons and brain stem, 3. Sagittal T1WI MRI showing thin corpus callosum, dilated lateral ventricle, and kinked brain stem with flat pons giving a Z-shaped brain stem, 4. Axial T2WI MRI showed abnormal cortical gyration in the form of anterior pachygyria/ lissencephaly, dilated lateral ventricles, and abnormality of the white matter. D) Patient 4 MRI images showing 1. Axial FLAIR showing extensive white matter affection, 2. Axial T2WI shows extensive white matter affection extending to the subcortical zone.

**SF 2** A representation of the locations in the proteins affected by the identified mutations in CMD patients. A POMGNT1 enzyme with a changed sequence of the active site in patient 1 and carbohydrate-binding stem domain in patient 3. B, laminin-α2 subunit with affected laminin EGF-like domains 10 and 15 for patients 2 and 4, respectively. The arrows point at the affected peptide sequences.

**SF 3** A portion of the sequencing electropherograms of *POMGNT1* showing the identified mutations. Arrow indicates the site of mutation.

**SF 4** Histogram charts of the quantification of western blot bands using image J software. A) Quantified bands of the α-DG showing relative expression of the intact protein in the control and its truncated forms in the studied patients. B) Quantified bands of LAMA2 protein showing its reduced expression in patients 1, 2, and 3 while it was expressed in patient 4 at levels comparable to the control.


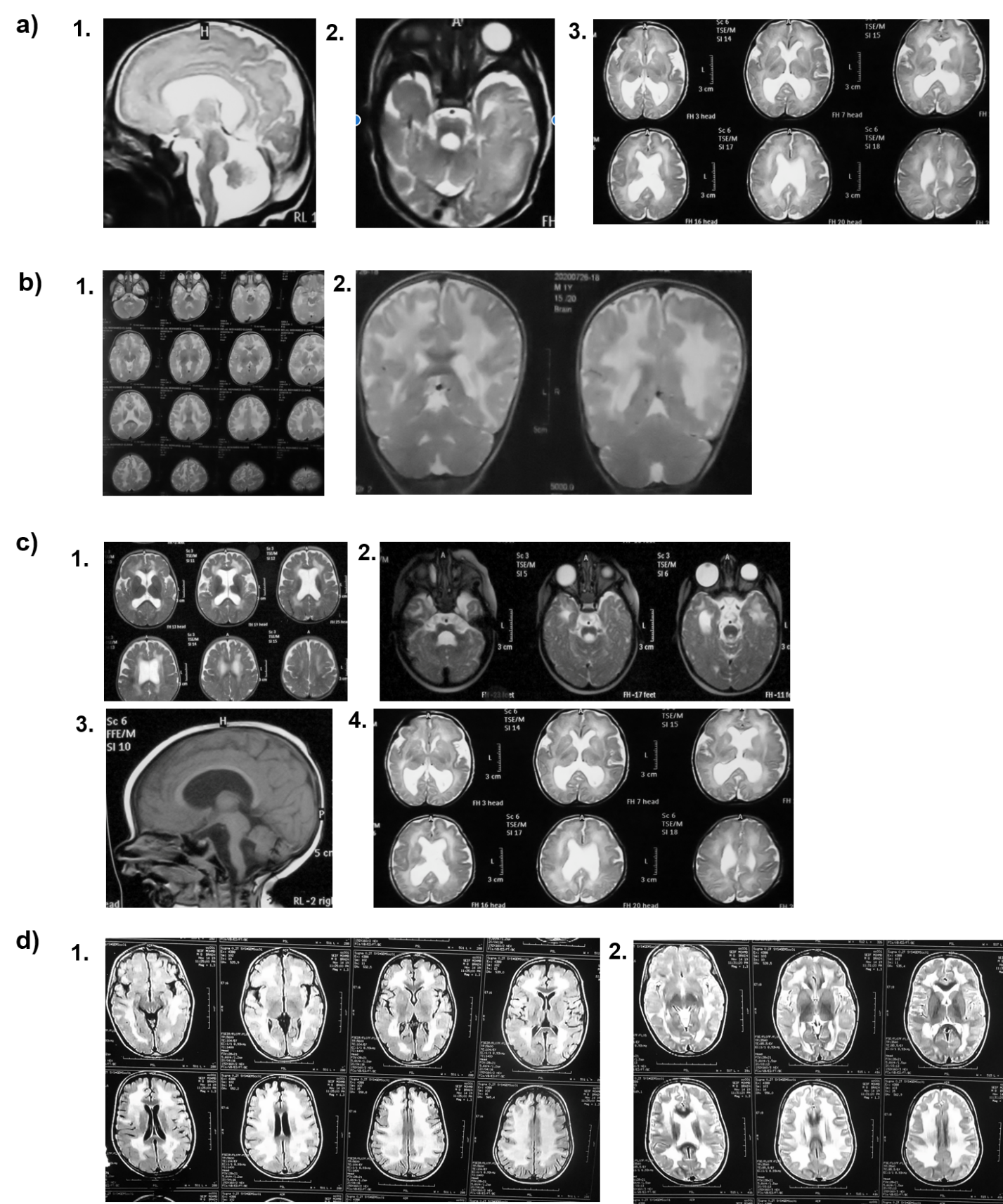


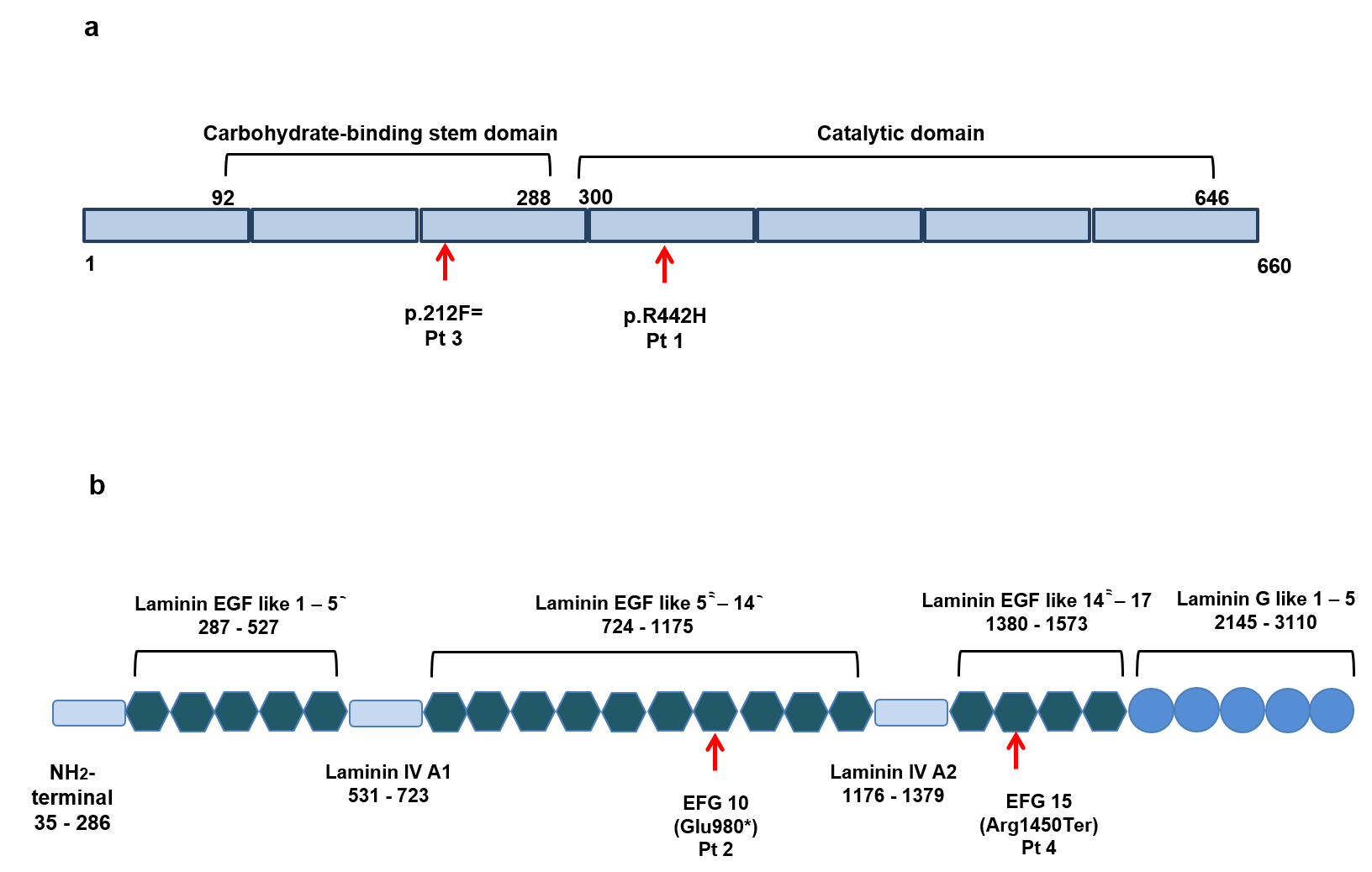


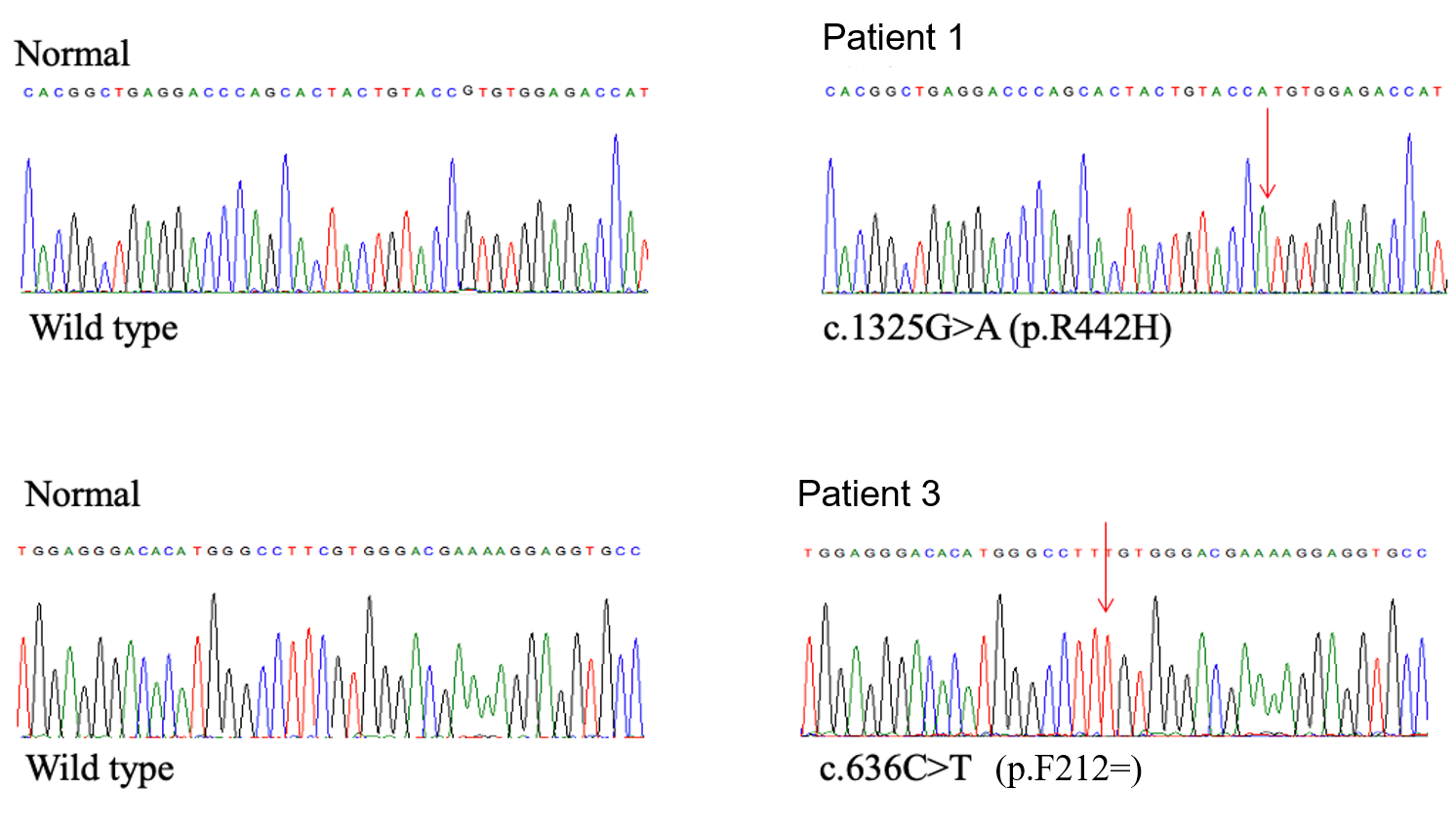


a)

b)


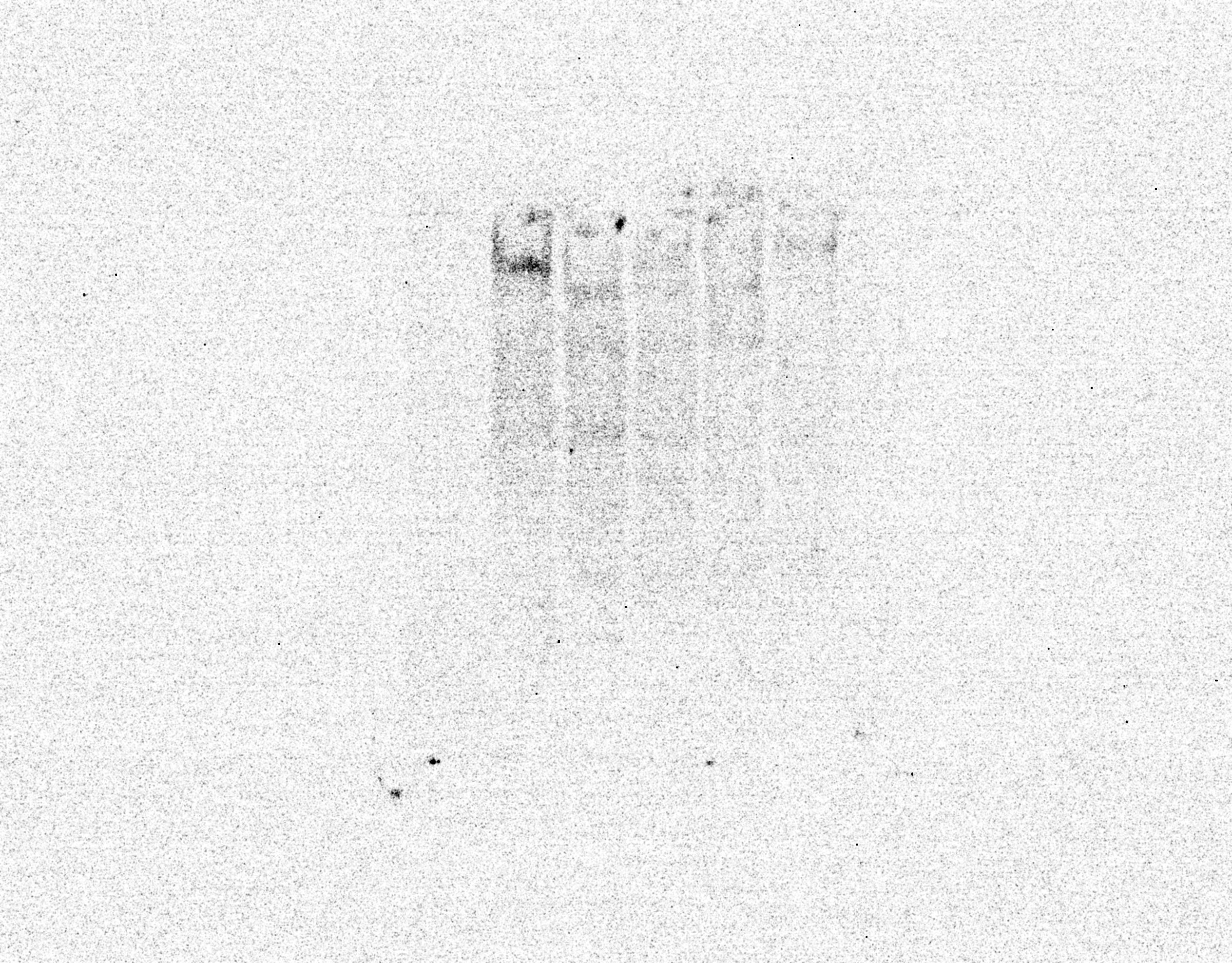


The original blot of figure 2a


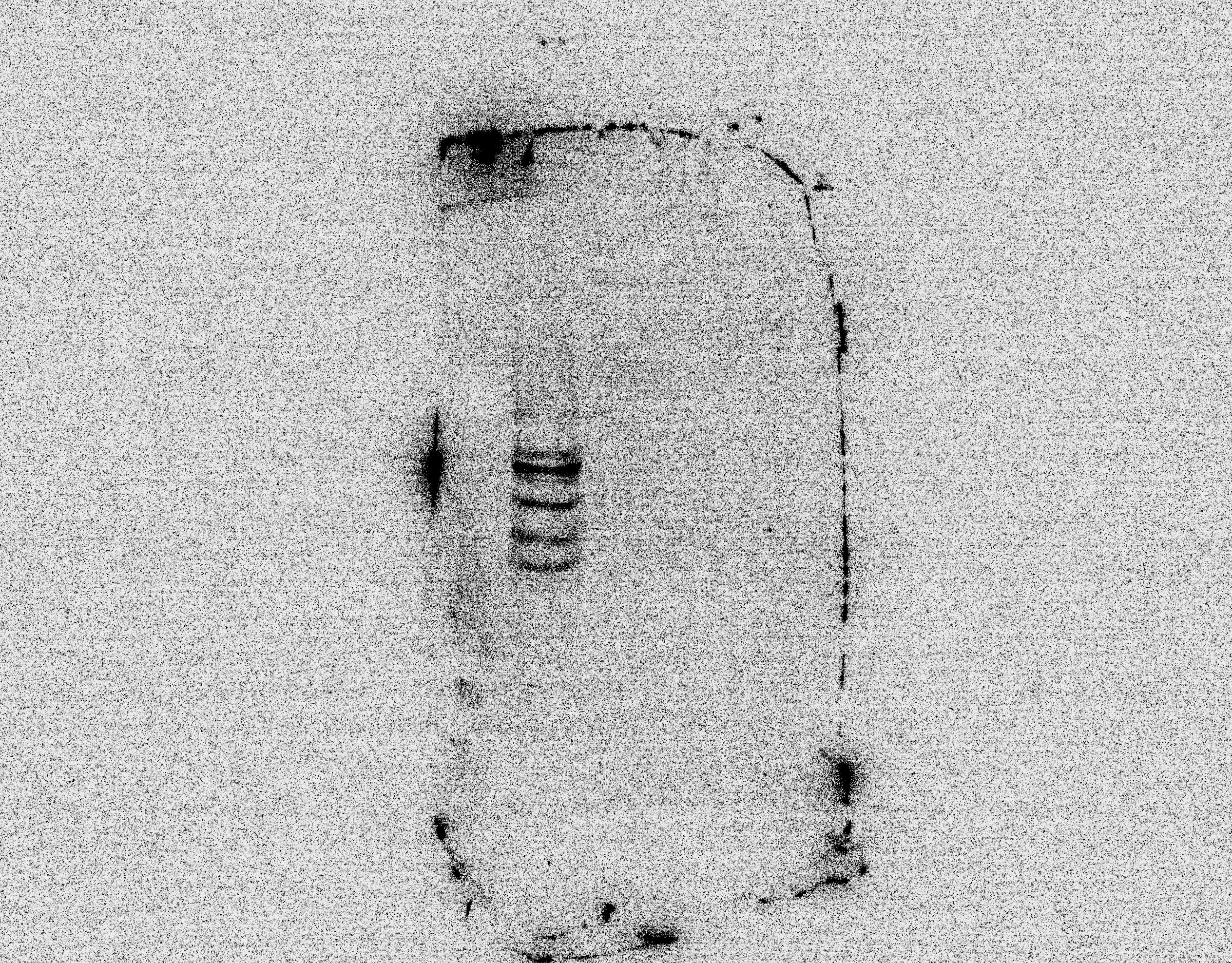


The original blot of figure 2b


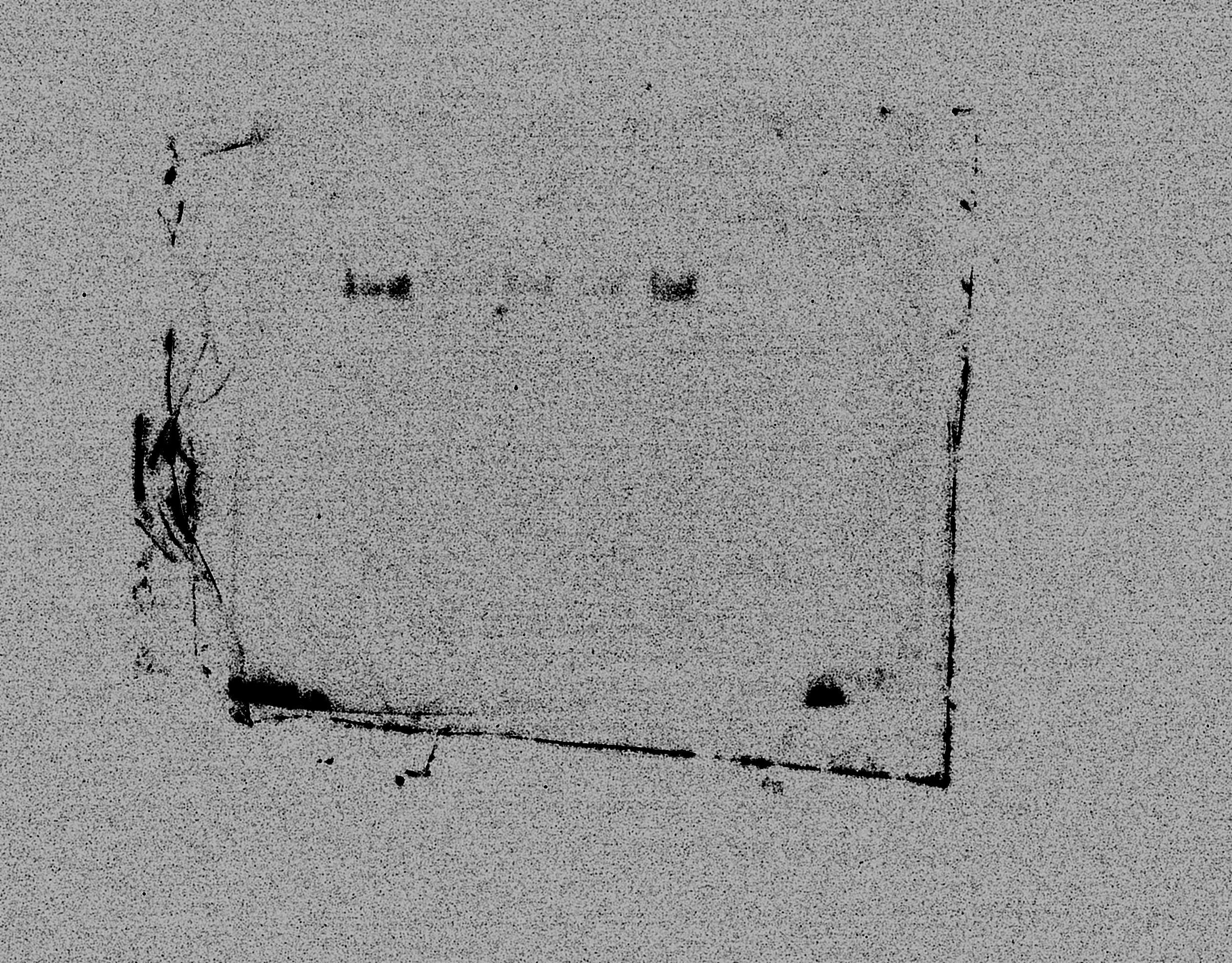


The original blot of figure 2c
